# The orofacial cleft risk gene *IRF6* is a target gene of SOX9 in cranial neural crest cells

**DOI:** 10.1007/s00109-026-02720-7

**Published:** 2026-09-26

**Authors:** Matthias Weider, Magdalena C. Wagner, Theresa Schmid, Sebastian Gehlen-Breitbach, Gabriele Rodrian, Nicolai Peschel, Holm Schneider, Kerstin U. Ludwig, Michael Wegner, Lina Gölz

**Affiliations:** 1 Zahnklinik 3 – Kieferorthopädie, Universitätsklinikum Erlangen and Friedrich-Alexander-Universität Erlangen-Nürnberg, Erlangen, Germany; 2 Institut Für Biochemie, Friedrich-Alexander-Universität Erlangen-Nürnberg, Erlangen, Germany; 3 Zentrum Für Ektodermale Dysplasien, Universitätsklinikum Erlangen, Erlangen, Germany; 4 Kinder- Und Jugendklinik, Universitätsklinikum Erlangen, Erlangen, Germany; 5 Institut Für Humangenetik, Medizinische Fakultät, Universitätsklinikum Bonn, Universität Bonn, Bonn, Germany

**Keywords:** Palate development, Craniofacial development, Cleft lip, Cleft palate, Transcriptional regulation, Craniofacial developmental biology, Single cell RNA-seq, Single cell transcriptomics, Cell-cell communication

## Abstract

**Abstract:**

Orofacial clefts represent the most prevalent congenital anomalies affecting the craniofacial region. They can be evoked by a disturbed development of either oral epithelium or cranial neural crest-derived mesenchyme, or by disruptions of their interplay. *IRF6* is a well-known risk gene associated with non-syndromic orofacial clefts and causative for Van der Woude syndrome, an autosomal dominant syndrome characterized by orofacial clefting and lower lip pits. Its expression in the oral epithelium depends on the transcription factor TFAP2A. We here show by immunofluorescence on mouse embryonic sections and by mining of single cell RNA-seq data that *IRF6* is also expressed in cranial neural crest-derived tissue in mice and humans, together with *TFAP2A* and *SOX9*. The *IRF6* enhancer MCS-9.7 is bound and activated by the transcription factor SOX9, mutations of which cause Pierre Robin sequence, a craniofacial anomaly that includes cleft palate. This SOX9-dependent activation is influenced by the single nucleotide variant rs76145088 that is associated with orofacial clefting. Inactivation of Sox9 in a murine cranial neural crest cell line by CRISPR/Cas9 results in loss of *Irf6* expression. We conclude that dysregulation of the SOX9–IRF6 axis in cranial neural crest cells could be relevant for the pathogenesis of orofacial clefting.

**Key Messages:**

- *Irf6* is co-expressed with *Sox9* – the major gene mutated in Pierre Robin Sequence (a craniofacial anomaly that includes cleft palate) – in a subset of cranial neural crest cells.

- The *IRF6* enhancer MCS-9.7 is bound and activated by SOX9 and different alleles of the orofacial cleft risk SNV rs76145088 in MCS-9.7 confer differential activation of this enhancer by SOX9.

- Inactivation of *Sox9* via CRISPR/Cas9 in a cranial neural crest cell line prevents *Irf6* expression.

**Supplementary Information:**

The online version contains supplementary material available at https://doi.org/10.1007/s00109-026-02720-7.

## Introduction

Orofacial clefts (OFC) comprise cleft lip and palate, isolated cleft lip and isolated cleft palate. These malformations represent some of the most prevalent congenital deformities in humans, with a global prevalence of approximately 1 in 600 births [1]. Non-syndromic OFC (nsOFC), which account for the majority of cases, are typically confined to the orofacial region without additional abnormalities. In contrast, syndromic OFC (sOFC) are associated with other congenital anomalies. While syndromic clefts typically exhibit monogenic inheritance, non-syndromic clefts are attributable to polygenic causes [2].

The aetiology and pathogenesis of OFC are highly complex, involving a combination of genetic and environmental factors [1]. Although many genetic risk factors have been identified for orofacial clefting, the specific cause and underlying mechanisms are still unknown in most individual cases [3–8]. These malformations occur during embryonic development, when the fusion of tissues in the facial region fails to proceed correctly. The development of the craniofacial region relies on ectodermal epithelial cells, on cranial neural crest cells (CNCCs) – which represent a transient, pluripotent stem cell-like cell population – and on the interactions of both cell types [9, 10]. Moreover, it depends on precisely coordinated gene regulatory networks that control key cellular activities such as differentiation, migration, proliferation and apoptosis. These processes drive growth, patterning and fusion of facial structures. Even minor disruptions can lead to congenital abnormalities such as OFC [11].

A critical factor in craniofacial development is Interferon Regulatory Factor 6 (*IRF6*). *IRF6* plays a pivotal role in craniofacial development, and mutations in its gene have been associated with both sOFC and nsOFC [7, 8, 12–14]. Causative *IRF6* variants are found in at least 70% of patients with Van der Woude Syndrome (VWS), the most common form of syndromic clefting, which follows an autosomal dominant inheritance pattern [13]. While causality of *IRF6* mutations for VWS is well established, pathogenic variants up to now have only been detected in < 1% of OFC patients [15]. Hence, *IRF6* should be considered as a gene that is associated with, i.e. attributing to OFC. IRF6 is a transcription factor that is primarily active in cells of epithelial origin during face formation and is essential for the proper fusion of facial structures [16]. It maintains the balance between differentiation and proliferation of the ectoderm during craniofacial development [16]. In *Irf6*-deficient mice, this balance is disrupted, resulting in a thickened and disorganized oral epithelium, impaired skin barrier function and early abnormal oral adhesions, ultimately leading to OFC [16, 17].

A key regulatory element in this process is the MCS-9.7 enhancer, which controls *IRF6* expression. Transcription factor AP2-α (TFAP2A) regulates MCS-9.7 activity, which in turn modulates *IRF6* expression during epidermal development [18, 19]. An allele of the single nucleotide variant (SNV) of rs642961 in MCS-9.7 disrupts TFAP2A binding, increasing the risk for nsOFC. The studies by Rahimov et al*.* and by others have helped to characterize the gene regulatory network involving *IRF6*, *TFAP2A*, and *GRHL3* [18–21]*,* a target gene of IRF6 in epithelial cells [22], and other target genes and upstream regulators of IRF6 [23, 24]. In addition to its well-established role during orofacial epithelial development [7, 25, 26], Irf6 was also detected in neuroectodermal structures, in subsets of early migrating neural crest cells (NCCs), in subsets of craniofacial chondrocytes and osteocytes and in tongue mesenchyme [20, 27–32]. As TFAP2A fulfils essential functions in NCCs and neural crest-derived structures [33, 34], we set out to further investigate the expression pattern of *IRF6* in CNCC-derived tissue as well as its upstream transcriptional regulators. We speculated that *SOX9* (SRY-box transcription factor 9) could be a relevant candidate. We came to this conclusion because *SOX9* is a *bona fide* cranial neural crest transcription factor [35] and is a causative gene for Pierre Robin sequence, a craniofacial anomaly that includes cleft palate. Moreover, rs76145088, a SNV in the vicinity of rs642961 that is also associated with nsOFC [18], is located within a DNA sequence that resembles a binding site for SOX9. Besides the analysis of the *IRF6* expression pattern, we therefore additionally analyzed the potential Sox9-dependent activation of MCS-9.7 and Sox9-dependent expression of *Irf6* in CNCCs.

## Results

### *Irf6* is co-expressed in cranial neural crest-derived tissue with* Tfap2a* and *Sox9*

**Fig. 1:**
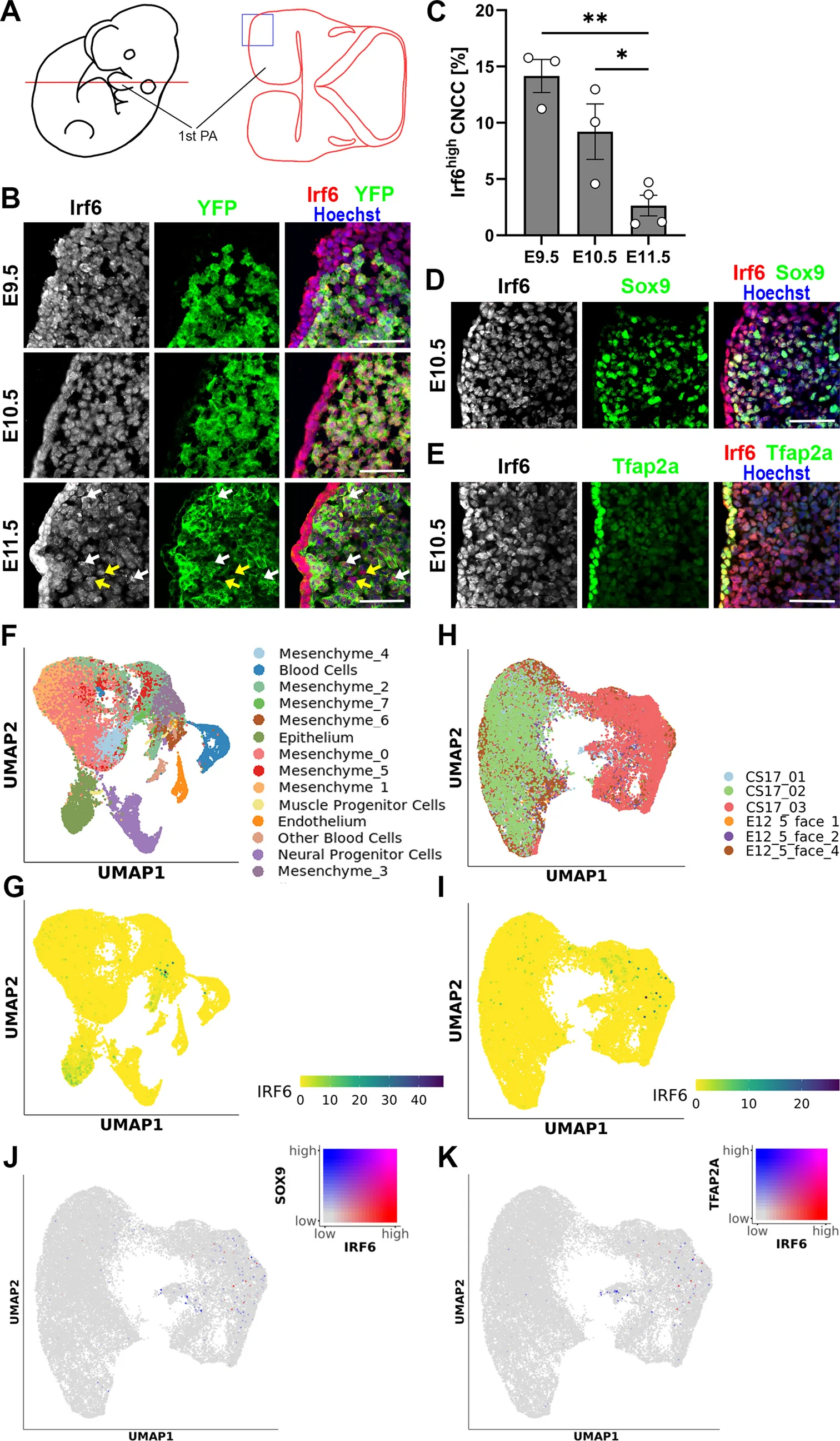
*Irf6* is co-expressed with *Sox9* and *Tfap2a* in cranial neural crest-derived mesenchymal tissue. (**A**) Schematic depicting the plane of section (red) within the first pharyngeal arch (PA) and the localization of panels within that plane as presented in B, D and E (blue square). (**B**) Immunofluorescent staining of Irf6 protein (red) neural crest-derived tissue of murine pharyngeal arches labelled by *Wnt1::Cre*-activated *YFP*-expression (green) from the *Rosa26-stopflox-YFP* locus at embryonic days E9.5, E10.5 and E11.5. Cell nuclei are counterstained with Hoechst 33342 (blue). White arrows point to Irf6^high^ CNCCs, yellow arrows to Irf6^high^ cells that are not neural-crest derived. (**C**) Percentage of Irf6^high^ CNCCs, defined as CNCCs displaying a mean Irf6 nuclear signal intensity equal to or higher than mean nuclear signal intensity in epithelial cells, at given embryonic stages. Statistical significance was determined by one-way ANOVA. Graph bars represent arithmetic means ± SEM (N ≥ 3). *0.01 ≤ *p* < 0.05**,** **0.001 ≤ *p* < 0.01. (**D, E**) Co-localization of Irf6 (red) with Sox9 (green, D) and Tfap2a (green, E). Cell nuclei are counterstained with Hoechst 33,342 (blue). (**F**) Uniform Manifold Approximation and Projection plots (UMAP) of all cell types from human CS17 and murine E12.5 craniofacial tissue calculated with scRNA-seq data from Yankee et al*.* 2023 [36]. (**G**) UMAP colored by normalized gene expression of *IRF6* on the dataset from (F). (**H**) UMAP of mesenchymal cells from human CS17 (CS17) and murine E12.5 (E12.5) craniofacial tissue calculated with scRNA-seq data from Yankee et al*.* 2023 [36]. (**I**) UMAP colored by normalized gene expression of *IRF6* on the dataset from (H). (**J, K**) UMAPs colored by normalized gene expression of *IRF6* (red) and *SOX9* (J, blue) or *TFAP2A* (K, blue) on the dataset from (H). Size bars: 50 µm

To obtain more insight into *Irf6-*expressing craniofacial tissues, we characterized *Irf6* expression by immunofluorescent staining in transgenic mice in which neural crest-derived tissue is labelled by expression of *YFP* due to a *Rosa26-stopflox-YFP* reporter activated by the *Wnt1::Cre* driver. Indeed, we detected Irf6-positive neural crest-derived cells at the analyzed stages E9.5, E10.5 and E11.5 (Fig. 1A, B). The Irf6 signal in mesenchymal tissue tendentially decreased with the course of development while the epithelial signal remained strong and even tended to increase (Fig. 1B, Fig. S1A). At E11.5, only a minor fraction of CNCCs (2.6% ± 0.9%) had retained a high Irf6 signal intensity equal to epithelial cells (Fig. 1C). We detected co-labelling of Irf6 with Sox9 in mesenchymal cells of the pharyngeal arches (Fig. 1D) and with Tfap2a predominantly in the epithelium (Fig. 1E). We tested specificity of the used anti-IRF6 antiserum by staining HaCaT cells transfected with shRNAs targeting human *IRF6*. Expectedly, the scrambled shRNA did not change nuclear signal intensity of IRF6 (Fig. S1B, C). Two shRNAs targeting *IRF6* decreased IRF6 signal intensity significantly, confirming specificity of the used antibody, while a third shRNA left IRF6 signal unchanged.

In order to gain further evidence for expression of *IRF6* in neural crest-derived tissue, we analyzed publicly available scRNA-seq data of embryonic craniofacial mouse tissue at E12.5 and human tissue at CS17 [36]. We detected *IRF6* in a subset of mesenchymal cells and – as expected – in the majority of epithelial cells (Fig. 1F, G, Fig. S1D). *IRF6*-positive cells were present both among human and murine mesenchymal cells in these embryonic samples (Fig. 1H, I). Many of the *IRF6*-positive mesenchymal cells were also positive for *SOX9* (56.0%, Fig. 1J, Supplementary Table 1) and for *TFAP2A* (86.8%, Fig. 1K, Supplementary Table 2). Expression of *IRF6* was specific for cells in two of seven mesenchymal Seurat clusters (Fig. S1E). These clusters were also positive for the majority of marker genes for osteogenic, odontogenic, perimysial, midline and mitotic CNCCs [37], preventing conclusions about the function of *IRF6*-positive cells.

### A SNV associated with risk for OFC influences SOX9-dependent activation of the *IRF6* enhancer MCS-9.7

**Fig. 2:**
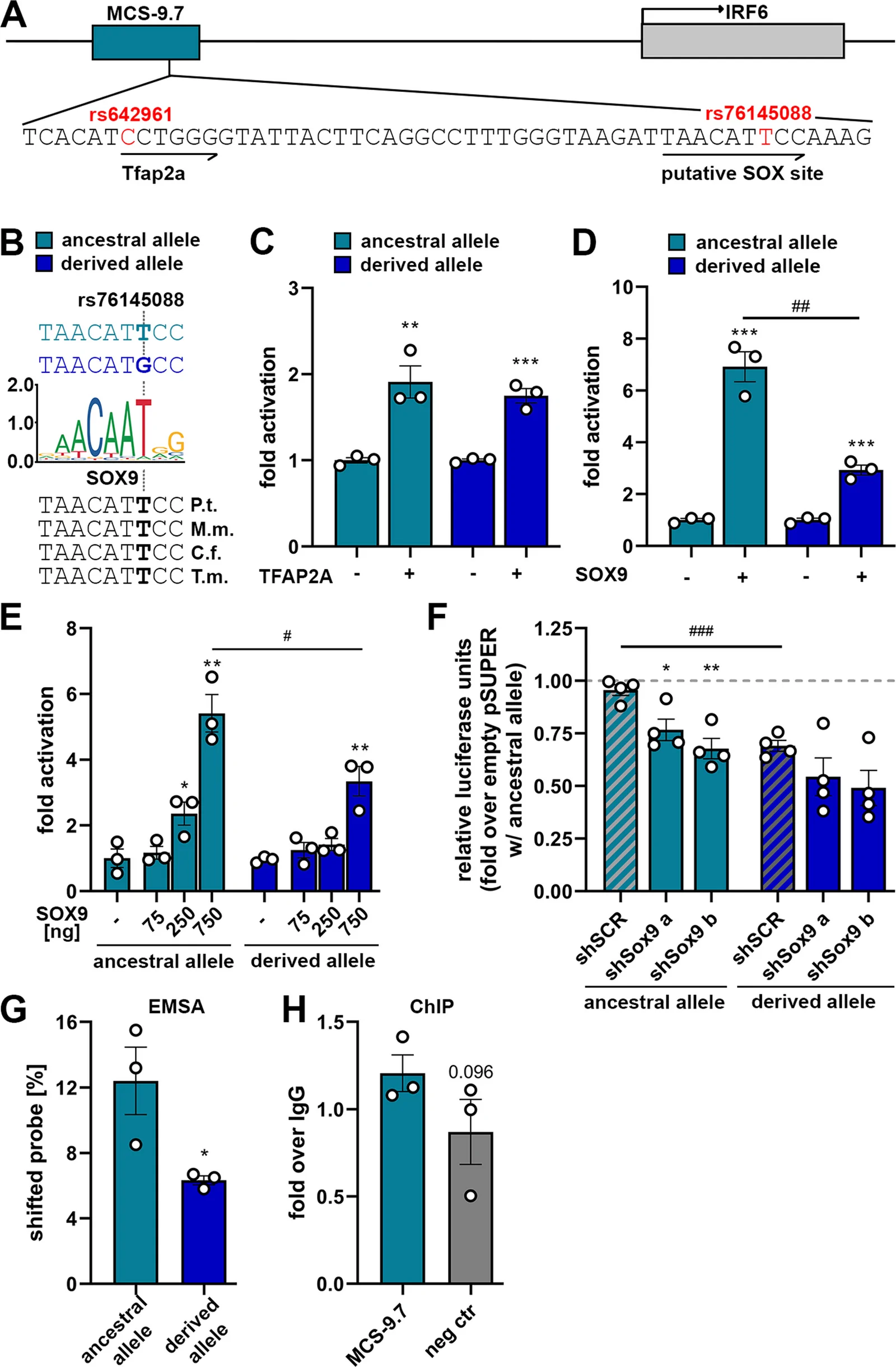
The SOX9-dependent activation of the *IRF6* enhancer MCS-9.7 is influenced by the orofacial cleft risk SNV rs76145088. (**A**) Schematic representation of *IRF6* and MCS-9.7 and zoom-in depicting nucleotide sequence with the TFAP2A binding site around rs64961 and the putative SOX binding site spanning rs76145088. (**B**) Sequences of putative SOX binding site around rs76145088 (in boldface) with ancestral allele (turquoise, upper line) and derived allele (blue, lower line) aligned to position weight matrix for SOX9 monomeric binding from JASPAR database (JASPAR release 2022, matrix ID MA0077.1) with sequence conservation in other species according to UCSC genome browser (P.t. chimp, M.m. mouse, C.f. dog, T.m. manatee). (**C, D**) Luciferase assays of MCS-9.7 containing ancestral (petrol bars) or derived allele (blue bars) of rs76145088 in Neuro-2a cells with effector plasmids expressing *Tfap2a* (+ in B) or *SOX9* (+ in C) or empty control vectors (-) with luciferase units of controls arbitrarily set to 1. (**E**) Luciferase assay of MCS-9.7 with varying amounts of SOX9 effector plasmid. (**F**) Luciferase assays of MCS-9.7 containing ancestral (petrol bars) or derived allele (blue bars) of rs76145088 in O9-1 cells co-transfected with shRNA-expressing pSUPER plasmids targeting murine Sox9 (shSox9 a, shSox9 b) or scrambled control (shSCR), with luciferase units of empty pSUPER co-transfected with ancestral allele arbitrarily set to 1 (indicated by grey stippled horizontal bar). (**G**) Quantification of oligonucleotide probe that is shifted by SOX9 in EMSA as percentage of whole probe signal intensity for ancestral allele (petrol bar) and derived allele (blue bar). (**H**) Quantification of precipitated chromatin from O9-1 cells with anti-Sox9 antiserum within the murine enhancer corresponding to MCS-9.7 (petrol bar) and of an upstream negative control region (grey bar), illustrated as enrichment of chromatin precipitated with anti-Sox9 antiserum over chromatin precipitated with IgG. Statistics were performed with t-tests between effectors and controls, between the different alleles or between PCR signal in MCS-9.7 vs. negative control, and with one-way ANOVA within each series (either ancestral or derived allele) containing samples with increasing or decreasing SOX9 amounts. Graph bars represent arithmetic means ± SEM (*N* ≥ 3). Asterisks depict statistically significant differences within experimental series containing a specific allelic variant of MCS-9.7, hashtags depict statistically significant differences between allelic variants. *0.01 ≤ *p* < 0.05, **0.001 ≤ *p* < 0.01, ***p < 0.001; ^#^0.01 ≤ *p* < 0.05, ^##^0.001 ≤ *p* < 0.01, ^###^*p* ≤ 0.001. Numbers above bars represent non-significant *p*-values

The *IRF6* enhancer MCS-9.7 contains a putative SOX transcription factor binding site (JASPAR release 2022, matrix ID MA0077.1) – although not perfectly aligning with the consensus sequence – in the vicinity of the TFAP2A binding site that is affected by rs642961 [18]. It caught our attention that the SOX9 consensus sequence spans rs76145088 (Fig. 2A, B), a SNV that was formerly named −14,474 [18], which is located on the same haplotype as rs642961 and is also associated with OFC [18]. This SOX9 consensus sequence is perfectly conserved in mammals (Fig. 2B) [38]. We therefore hypothesized that MCS-9.7 might be activated by SOX9. To test this hypothesis, we cloned MCS-9.7 [18] into a luciferase reporter plasmid upstream of a minimal promoter. We cloned the ancestral allele, which represents the OFC risk allele, as well as the derived allele of rs76145088 (Fig. 2B). As a proof of principle, we tested activation of this enhancer by TFAP2A [18]. Indeed, we could recapitulate the reported TFAP2A-dependent activation of MCS-9.7 by luciferase assays in the neural crest-derived cell line Neuro-2a where TFAP2A activated MCS-9.7 (Fig. 2C). The derived allele of rs76145088 did not influence activation by TFAP2A, as there was no difference in activation of both alleles (Fig. 2C).

SOX9 was also able to activate MCS-9.7 (Fig. 2D). Strikingly, the derived allele of rs76145088 caused a drastically reduced activation by SOX9 compared with the ancestral allele. In line with this finding, the guanine base of the derived allele does not match the SOX9 binding consensus (Fig. 2B). By varying the amount of effector plasmid, we could show that the derived allele needs higher SOX9 levels to be activated: while already 250 ng of effector plasmid led to significant activation of MCS-9.7 with sequence of the ancestral allele, 750 ng were needed for activation of the MCS-9.7 variant with sequence of the derived allele (Fig. 2E).

In order to test whether MCS-9.7 is activated by endogenous Sox9 levels in CNCC-derived cells, we co-transfected the murine CNCC line O9-1 [39] with the luciferase reporters and with pSUPER plasmids containing shRNAs targeting murine *Sox9* (shSox9 a, shSox9 b), with scrambled control (shSCR) or with empty pSUPER. Effective knockdown of Sox9 protein levels had been confirmed beforehand (Fig. S2A). While the scrambled control did not influence luciferase activity, each shRNA targeting *Sox9* expectedly reduced activity of the ancestral allele of MCS-9.7 (Fig. 2F). Intriguingly, the derived allele of rs76145088 (co-transfected with shSCR, i.e. without reduction of Sox9 levels) evoked a significantly weaker activation than the ancient allele. The shRNAs targeting *Sox9* further reduced luciferase units of the derived allele slightly, but not statistically significant. We conclude that MCS-9.7 is activated by endogenous Sox9 in CNCCs and that this activation is influenced by rs76145088.

We analyzed binding of SOX9 to the putative binding site spanning rs76145088 by electrophoretic mobility shift assay (EMSA). Indeed, this site was bound by SOX9 (Fig. 2G, Fig. S2B). In accordance to the stronger SOX9-dependent activation of the ancestral allele, SOX9 binding to this allele was more robust than to the derived allele (Fig. 2G, Fig. S2B). We asked whether SOX9 interacts with this DNA element in CNCCs. To this aim, we performed chromatin immunoprecipitation (ChIP) in O9-1 cells. With antiserum directed against Sox9, we precipitated tendentially more chromatin from the region corresponding to human MCS-9.7 than from an upstream negative control region (Fig. 2H), suggesting a weak binding of Sox9 to this element in CNCCs.

### Expression of Irf6 in CNCCs depends on Sox9

**Fig. 3:**
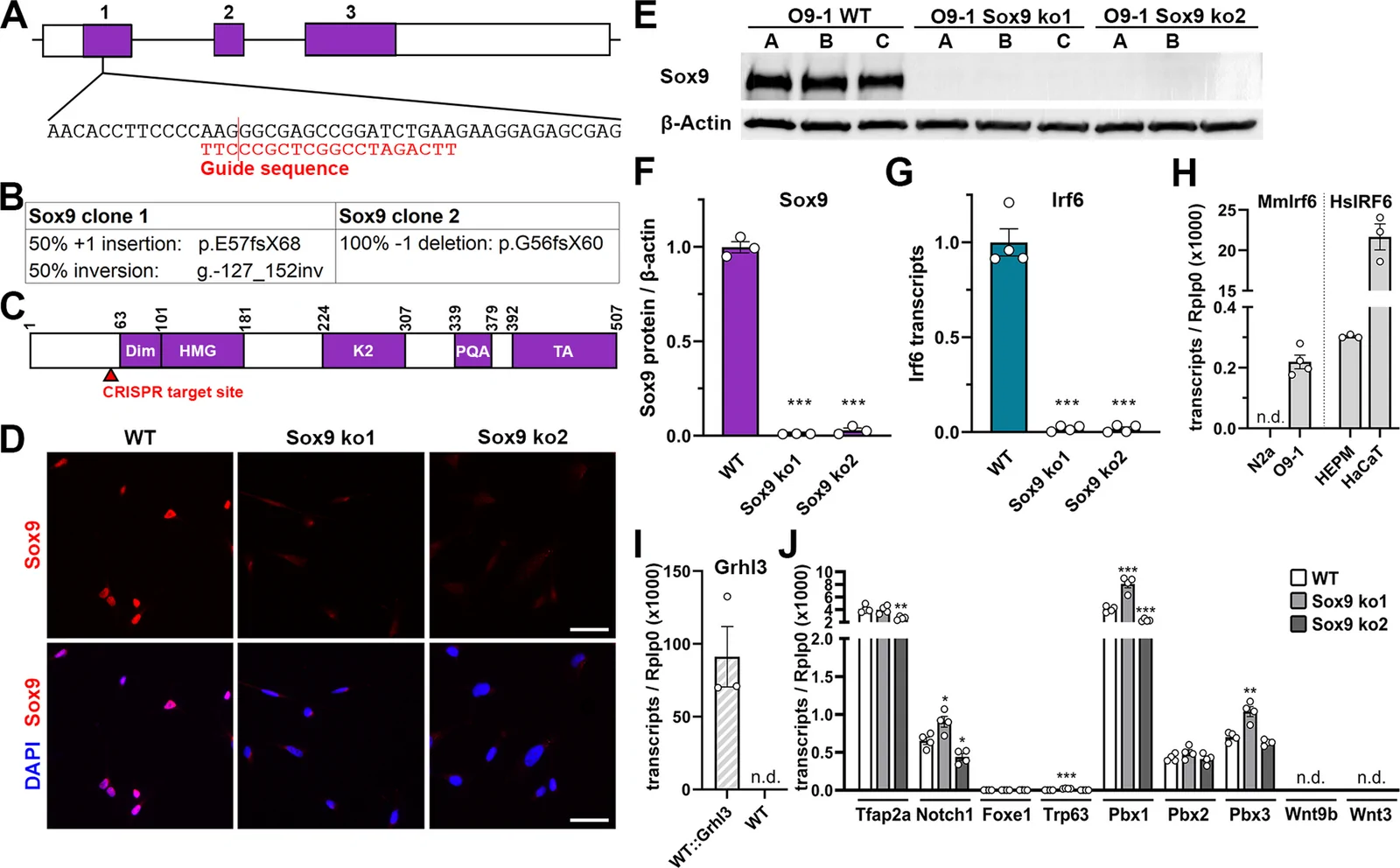
Expression of *Irf6* in O9-1 cranial neural crest cells depends on Sox9. (**A**) Schematic representation of Sox9 protein and genomic sequence around start ATG with CRISPR/Cas9 guide sequence (red letters). (**B**) Characterization of CRISPR/Cas9-induced gene editing events in Sox9 knockout clones. (**C**) Schematic representation of Sox9 protein with functionally relevant domains and their amino acid positions (Dim, dimerization domain; HMG, high mobility group DNA binding domain; K2 conserved domain K2; PQA, glutamine-rich domain; TA transactivation domain) and localization of the site targeted by CRISPR/Cas9. (**D**) Immunofluorescent staining of Sox9 (red) in WT O9-1 cells and Sox9-edited clones (Sox9 ko1, Sox9 ko2). DAPI served as nuclear counterstain (blue). (**E**) Western blot of Sox9 and beta-actin proteins in WT O9-1 cells and Sox9-edited clones in three independent samples each. (**F**) Quantification of Sox9 protein levels relative to beta-actin levels ± SEM as determined by Western blot. Sox9 levels in WT cells were arbitrarily set to 1. Statistical significance was determined by comparing Sox9 levels in each ko clone with WT by t-test (*N* = 3). (**G**) Quantification of *Irf6* transcript levels ± SEM in WT O9-1 cells and Sox9 ko clones as determined by qRT-PCR relative to *Rplp0*. *Irf6* transcript levels in WT cells were arbitrarily set to 1. Statistical significance was determined by comparing *Irf6* transcript levels in each ko clone with WT by t-test (*N* = 4). (**H**) Quantification of *Irf6* transcript levels ± SEM in neural-crest derived (Neuro-2a = N2a, O9-1, HEPM) and epithelial cell lines (HaCaT) as determined by qRT-PCR relative to *Rplp0* (*N* ≥ 3). Primers were designed for murine *Irf6* and *Rplp0* or human *IRF6* and *RPLP0* depending on the species of the cell line as indicated. (**I**) Quantification of *Grhl3* transcript levels ± SEM in O9-1 cells (WT) determined by qRT-PCR relative to *Rplp0* (N = 3). O9-1 cells that had been transfected with *Grhl3* expression plasmid served as positive control (WT::Grhl3). (**J**) Quantification of *Tfap2a*, *Notch1*, *Foxe1*, *Trp63*, *Pbx1*, *Pbx2*, *Pbx3*, *Wnt9b* and *Wnt3* transcript levels ± SEM in WT O9-1 cells (white bars) and Sox9 ko clones (light grey and dark grey bars) as determined by qRT-PCR relative to *Rplp0*. Statistical significance was determined by comparing transcript levels in each ko clone with WT by t-test (*N* = 4). Size bar: 50 µm; *0.01 ≤ *p* < 0.05, **0.001 ≤ *p* < 0.01, ***p < 0.001; n.d. = not detected

The overlapping expression pattern of *IRF6* and *SOX9* and the activation of MCS-9.7 by SOX9 tempted us to speculate that *IRF6* expression in CNCCs might depend on SOX9. To test this hypothesis, we inactivated *Sox9* in O9-1 cells via CRISPR/Cas9. We targeted nucleotide position 149 (mm10:chr11:112,782,750) of the murine *Sox9* coding sequence (Fig. 3A). One obtained clone possessed a −1 frameshift at the corresponding amino acid glycine 56 of Sox9 (Fig. 3B) in all alleles. Another clone displayed a + 1 frameshift at the equivalent glutamate 57 in half the alleles while the other half displayed drastic rearrangements around the *Sox9* start codon with an inversion of a 279 bp fragment (Fig. 3B). These mutations are predicted to prevent translation or would only allow the generation of substantially shortened Sox9 proteins without functionally essential domains (Fig. 3C). According to immunofluorescent stainings (Fig. 3D) and Western blot (Fig. 3E, F), Sox9 protein was completely absent in both clones.

After successful generation of two independent *Sox9*-deficient O9-1 clonal lines, we tested if *Irf6* expression is affected in these cells. To this aim, we extracted RNA from proliferating cells and performed quantitative reverse transcriptase PCR (qRT-PCR). Indeed, *Irf6* transcript levels were virtually absent in both *Sox9* ko clones (Fig. 3G). We conclude that expression of *Irf6* in CNCCs depends on Sox9.

In order to evaluate expression levels of *Irf6* in O9-1 cells, we compared transcript levels in neural crest-derived O9-1, Neuro-2a and HEPM cells with levels in epithelial HaCaT cells (Fig. 3H). While *Irf6* was absent from Neuro-2a cells, we detected a weak, but reliable expression of *Irf6* in both craniofacial mesenchymal cell lines O9-1 and HEPM. In line with *Irf6* expression levels, activity of MCS-9.7 was weaker in O9-1 than in HaCaT cells (Fig. S3). We analyzed expression of the known *Irf6* target gene *Grhl3*, but could not detect any transcript in O9-1 cells (Fig. 3I). Finally, we asked whether Sox9 might exert its effect on *Irf6* expression also via other upstream regulators. Therefore, we analyzed expression of the known upstream regulators *Tfap2a*, *Notch1*, *Foxe1*, *Trp63*, *Pbx1*, *Pbx2*, *Pbx3*, *Wnt9b* and *Wnt3* in WT O9-1 cells and the Sox9-deficient clones. We only detected mild gene expression changes that were never consistent for any upstream regulator in both Sox9 knockout clones (Fig. 3J), arguing for a direct role of Sox9 in *Irf6* transcriptional regulation.

## Discussion

*IRF6* is a long-known risk factor for both sOFC and nsOFC [12, 13]. This has been attributed to the function of IRF6 in oral epithelial cells, specifically the periderm [18, 20, 22, 25, 26]. Nevertheless, some reports described expression of *Irf6* in neural crest-derived cells: Irf6 protein could be co-localized with Tfap2a in early migrating NCCs in mouse embryos at E9.5 [20] and *Irf6* transcripts were identified by RNAscope in migratory NCCs at E9.5 and in neural crest-derived craniofacial mesenchyme at E13.5 [27]. Additionally, Irf6 was detected in subsets of craniofacial chondrocytes and osteocytes of mouse embryos [30] and in pharyngeal arches and ethmoid plate chondrocytes of zebrafish embryos [28]. Moreover, abnormal bone formation in *Irf6* null mutants, especially in the craniofacial region [17], points to mesenchymal functions of *Irf6.* Here, we complement these findings by detection of Irf6 protein in murine neural crest-derived craniofacial mesenchyme labelled by *Rosa26-stopflox-YFP X Wnt1::Cre* from E9.5 to E11.5. Moreover, we detected co-expression of *Sox9* and *Tfap2a* with *Irf6* in these cells. It should be noted that *SOX9* is the major gene mutated in patients with Pierre Robin sequence, a craniofacial anomaly comprising mandibular hypoplasia and retrognathia, often in combination with glossoptosis and cleft palate [40]. By data mining of embryonic orofacial scRNA-seq experiments, we detected a fraction of orofacial mesenchymal cells that is positive for *IRF6* both in humans and mice. The majority of *IRF6*-positive cells were also positive for *SOX9*, both in mouse tissue as well as in human and mouse craniofacial mesenchymal cells. This correlates with the finding that SOX9 can activate the *IRF6* enhancer MCS-9.7. Although MCS-9.7 is primarily active in ectodermal structures [18], this enhancer possesses some activity in the pharyngeal arches [18, 41], which is consistent with its activation by the neural crest-specific Sox9. It should be noted that we detected significantly lower *Irf6* expression and MCS-9.7 activity in CNCC-derived O9-1 cells than in ectodermal HaCaT cells. Remarkably, MCS-9.7 activity and *Irf6* expression were also detected in cartilage and in tongue mesenchyme, tissues in which *Sox9* is also expressed [31, 32]. Importantly, we were able to show that Sox9 is necessary for expression of *Irf6* in the CNCC line O9-1. Known upstream regulators of *Irf6* were not substantially deregulated in Sox9 mutant clones, implying that regulation by Sox9 is direct. Direct activation of MCS-9.7 by Sox9 in CNCCs can also be inferred from the decreased activation resulting from reduced Sox9 levels, in conjunction with EMSA and ChIP results. We did not detect expression of *Grhl3* in O9-1 cells, suggesting that relevant target genes of Irf6 in CNCCs differ from epithelial cells [22].

The SNV rs76145088 that is associated with orofacial clefting is located in MCS-9.7 [18]. We identified a putative SOX transcription factor binding site spanning rs76145088, although not perfectly aligned with the consensus. SOX transcription factors often bind sequences not perfectly resembling consensus sequences [42]. Intriguingly, SOX9-dependent activation of MCS-9.7 was influenced by this SNV: the ancestral allele conveyed a more robust binding of SOX9 and thus a stronger activation by SOX9 than the derived allele, in line with the sequence similarity to the SOX9 consensus motif and the conservation of this sequence in mammals. This conservation might be attributable to the function of MCS-9.7 in epithelial cells and independent of Sox9. The fact that reducing Sox9 levels did not significantly reduce luciferase units of the derived allele is in line with our hypothesis that the SOX binding site around rs76145088 is essential for Sox9-depenedent activation. The slight reduction of luciferase levels and the residual enhancer activity hint to additional Sox9 binding sites within MCS-9.7. Multiple binding sites for SOX proteins in enhancers is a very common phenomenon [42].

Interestingly, the ancestral allele of rs76145088 that conveys stronger SOX9-dependent activation is the one that is associated with the risk for orofacial clefting. OFC might therefore be evoked by prolonged or excessive expression of *IRF6* in craniofacial mesenchymal cells – by an alternative mechanism to the many known *IRF6* mutations in epithelial cells that cause reduced expression or loss-of-function [7, 8, 13, 18]. An example for such a gain-of-function scenario is the finding that increased binding affinity of the transcription factor ETS1 to an enhancer of *SHH* causes polydactyly [43]. The fact that the risk allele of rs76145088 is the ancestral allele and more common in the first described Iowa population [18] as well as in each population listed in the UCSC Genome Browser [38] might be surprising at first glance, as OFC – although one of the most common birth defects – occur only with a frequency of 1:600 [1]. The seeming discrepancy can be accounted for by the multifactorial threshold model of inheritance for nsOFC [44]. While rs76145088 effects expression of *Irf6* in CNCCs, it might have no relevance for OFC but rather another SNV in 1q32. Deletion of *Irf6* in migratory NCCs via *Sox10::Cre* yielded no phenotypic alterations while deletion in pre-migratory NCCs via *Wnt1::Cre2* lead to decreased mineralization of the frontal and parietal bone [27]. Palatal defects were not observed, but a high perinatal lethality was reported [27].According to the LDlink database at NIH (https://ldlink.nih.gov/ldpair), rs76145088 is not in linkage disequilibrium with rs642961. We therefore speculate that it is not so much the ancestral allele of rs76145088 that has a risk effect, but rather that the derived allele has a protective effect (in conjunction with the G allele of rs642961). Of note, because transcription factors such as IRF6 and SOX9 are important regulators of cell function and fate, their levels have to be tightly regulated. Accordingly, not only decreased expression but also increased expression can lead to various drastic effects including OFC [42, 45]. As a matter of fact, overexpression of *Irf6* in the neural tube causes exencephaly [20] and an *IRF6* gain-of-function variant apparently causes ectodermal dysplasia [46]. It is therefore conceivable that prolonged or excessive expression of *IRF6* in CNCCs – in combination with other influences – can lead to occurrence of OFC.

It must be stated that other literature about Irf6 often detects immunofluorescent signal in the cytoplasm rather than a nuclear staining. This might be attributable to differing epitopes that could be masked by certain protein modifications. Such masked epitopes could also explain why other studies did not detect *Irf6* expression in palatal mesenchyme [32]. We verified specificity of the antibody by knockdown experiments in HaCaT cells. In any case, a nuclear localization fits well to the function of Irf6 as a transcription factor.

The fact that only a minor fraction of CNCCs expresses *IRF6* is in accordance with the presented scRNA-seq data [36] at the stages E12.5 in mouse and CS17 in human and with published data of Irf6 stainings on mouse and zebrafish tissue [20, 27–30]. Moreover, it might explain the variable phenotypic severity of midline cranial defects and variable perinatal lethality of homozygous conditional mutant mouse embryos in which *Irf6* had been deleted with *Wnt1::Cre2* [27]. Interestingly, MCS-9.7 displays both active and repressive histone modifications in NCCs and in craniofacial tissue [47]. This is typical for a poised enhancer, but would also be obtained in case of CNCC heterogeneity, when a subset of cells carries the active marks – causing *IRF6* expression – and the other cells the repressive marks – keeping the enhancer in a silent state. This, together with the low expression of *Irf6* in O9-1 cells may account for the finding that we were only able to detect a moderate enrichment of precipitated chromatin of the enhancer with an anti-Sox9 antiserum, as these facts lead to the expectation that the enhancer is only rarely bound by Sox9. The heterogeneity in mesenchymal craniofacial cells might be a research area worth investigating in future studies.

In summary, we suggest that IRF6 possesses a yet to be identified function in a subset of CNCCs. The expression of *IRF6* in these cells depends on SOX9 with SOX9-dependent activation of MCS-9.7 being higher in the risk allele of rs76145088. We conclude that dysregulation of the SOX9–IRF6 axis in CNCCs might be relevant for the pathogenesis of orofacial clefting.

## Materials & methods

### Mouse husbandry

Mouse housing and experiments were performed in accordance with animal welfare laws and all relevant ethical regulations. They were approved by the responsible government bodies and local committees (Regierung von Unterfranken, Veterinäramt der Stadt Erlangen). Mice carried a *Wnt1::Cre* transgene [48] as driver for neural crest-specific activation of a *Rosa26-stopflox-YFP* allele [49]. Genotyping was performed as described before [48, 49]. Mice were on a mixed C3H x C57Bl/6-J background and kept with continuous access to food and water under standard housing conditions in 12:12 h light–dark cycles.

### Cell culture

The O9-1 cell line was purchased from Sigma-Aldrich and cultured essentially as described [39, 50] and as briefly outlined in the supplementary methods section, as is culture of HaCaT cells (CLS GmbH), HEK293T cells (DSMZ) and Neuro-2a cells (DSMZ).

### Cloning procedures, Luciferase assays, Generation of CRISPR/Cas9 knockout clones, Immunofluorescent stainings, RNA extraction, qRT-PCR, Western blot, EMSA and ChIP

The listed methods were performed as outlined in the supplementary methods section.

## Supplementary Information

Below is the link to the electronic supplementary material.

Supplementary file1 (PDF 114 KB)

Supplementary file2 (XLSX 19 KB)

Supplementary file3 (TIF 1207 KB)

Supplementary file4 (TIF 729 KB)

Supplementary file5 (TIF 105 KB)

Supplementary file6 (TIF 1182 KB)

Supplementary file7 (TIF 1439 KB)

## Data Availability

All data are provided in the paper and its supplement.

## References

[1] Mossey PA, Little J, Munger RG, Dixon MJ, Shaw WC (2009) Cleft lip and palate. Lancet (London, England) 374:1773–1785 10.1016/S0140-6736(09)60695-4 19747722

[2] Stuppia L, Capogreco M, Marzo G, La Rovere D, Antonucci I, Gatta V, Palka G, Mortellaro C, Tete S (2011) Genetics of syndromic and nonsyndromic cleft lip and palate. J Craniofac Surg 22:1722–1726 10.1097/SCS.0b013e31822e5e4d 21959420

[3] Dixon MJ, Marazita ML, Beaty TH, Murray JC (2011) Cleft lip and palate: understanding genetic and environmental influences. Nat Rev Genet 12:167–178 10.1038/nrg2933 PMC3086810 21331089

[4] Wilson K, Newbury DF, Kini U (2023) Analysis of exome data in a UK cohort of 603 patients with syndromic orofacial clefting identifies causal molecular pathways. Hum Mol Genet 32:1932–1942 10.1093/hmg/ddad023 PMC10196673 37010288

[5] Birnbaum S, Ludwig KU, Reutter H, Herms S, Steffens M, Rubini M, Baluardo C, Ferrian M, Almeida de Assis N, Alblas MA et al (2009) Key susceptibility locus for nonsyndromic cleft lip with or without cleft palate on chromosome 8q24. Nat Genet 41:473–477 10.1038/ng.333 19270707

[6] Mangold E, Ludwig KU, Birnbaum S, Baluardo C, Ferrian M, Herms S, Reutter H, de Assis NA, Chawa TA, Mattheisen M et al (2010) Genome-wide association study identifies two susceptibility loci for nonsyndromic cleft lip with or without cleft palate. Nat Genet 42:24–26 10.1038/ng.506 20023658

[7] Rahimov F, Nieminen P, Kumari P, Juuri E, Nikopensius T, Paraiso K, German J, Karvanen A, Kals M, Elnahas AG et al (2024) High incidence and geographic distribution of cleft palate in Finland are associated with the IRF6 gene. Nat Commun 15:9568 10.1038/s41467-024-53634-2 PMC11538390 39500877

[8] Kumari P, Friedman RZ, Curtis SW, Pi L, Paraiso K, Visel A, Rhea L, Dunnwald M, Patni AP, Mar D et al (2025) Identification of functional non-coding variants associated with orofacial cleft. Nat Commun 16:6545 10.1038/s41467-025-61734-w PMC12267437 40670354

[9] Roth DM, Bayona F, Baddam P, Graf D (2021) Craniofacial development: neural crest in molecular embryology. Head Neck Pathol 15:1–15 10.1007/s12105-021-01301-z PMC8010074 33723764

[10] Meng L, Bian Z, Torensma R, Von den Hoff JW (2009) Biological mechanisms in palatogenesis and cleft palate. J Dent Res 88:22–33 10.1177/0022034508327868 19131313

[11] Wilkie AO, Morriss-Kay GM (2001) Genetics of craniofacial development and malformation. Nat Rev Genet 2:458–468 10.1038/35076601 11389462

[12] Zucchero TM, Cooper ME, Maher BS, Daack-Hirsch S, Nepomuceno B, Ribeiro L, Caprau D, Christensen K, Suzuki Y, Machida J et al (2004) Interferon regulatory factor 6 (IRF6) gene variants and the risk of isolated cleft lip or palate. N Engl J Med 351:769–780 10.1056/NEJMoa032909 15317890

[13] Kondo S, Schutte BC, Richardson RJ, Bjork BC, Knight AS, Watanabe Y, Howard E, de Lima RL, Daack-Hirsch S, Sander A et al (2002) Mutations in IRF6 cause Van der Woude and popliteal pterygium syndromes. Nat Genet 32:285–289 10.1038/ng985 PMC3169431 12219090

[14] Rojas-Martinez A, Reutter H, Chacon-Camacho O, Leon-Cachon RB, Munoz-Jimenez SG, Nowak S, Becker J, Herberz R, Ludwig KU, Paredes-Zenteno M et al (2010) Genetic risk factors for nonsyndromic cleft lip with or without cleft palate in a Mesoamerican population: evidence for IRF6 and variants at 8q24 and 10q25. Birth Defects Res A Clin Mol Teratol 88:535–537 10.1002/bdra.20689 20564431

[15] Leslie EJ, Koboldt DC, Kang CJ, Ma L, Hecht JT, Wehby GL, Christensen K, Czeizel AE, Deleyiannis FW, Fulton RS et al (2016) IRF6 mutation screening in non-syndromic orofacial clefting: analysis of 1521 families. Clin Genet 90:28–34 10.1111/cge.12675 PMC4783275 26346622

[16] Richardson RJ, Dixon J, Malhotra S, Hardman MJ, Knowles L, Boot-Handford RP, Shore P, Whitmarsh A, Dixon MJ (2006) Irf6 is a key determinant of the keratinocyte proliferation-differentiation switch. Nat Genet 38:1329–1334 10.1038/ng1894 17041603

[17] Ingraham CR, Kinoshita A, Kondo S, Yang B, Sajan S, Trout KJ, Malik MI, Dunnwald M, Goudy SL, Lovett M et al (2006) Abnormal skin, limb and craniofacial morphogenesis in mice deficient for interferon regulatory factor 6 (Irf6). Nat Genet 38:1335–1340 10.1083/ng1903 PMC2082114 17041601

[18] Rahimov F, Marazita ML, Visel A, Cooper ME, Hitchler MJ, Rubini M, Domann FE, Govil M, Christensen K, Bille C et al (2008) Disruption of an AP-2alpha binding site in an IRF6 enhancer is associated with cleft lip. Nat Genet 40:1341–1347 10.1038/ng.242 PMC2691688 18836445

[19] Kousa YA, Fuller E, Schutte BC (2018) IRF6 and AP2A interaction regulates epidermal development. J Invest Dermatol 138:2578–2588 10.1016/j.jid.2018.05.030 PMC12959130 29913133

[20] Kousa YA, Zhu H, Fakhouri WD, Lei Y, Kinoshita A, Roushangar RR, Patel NK, Agopian AJ, Yang W, Leslie EJ et al (2019) The TFAP2A-IRF6-GRHL3 genetic pathway is conserved in neurulation. Hum Mol Genet 28:1726–1737 10.1093/hmg/ddz010 PMC6494790 30689861

[21] Mangold E, Böhmer AC, Ishorst N, Hoebel AK, Gültepe P, Schuenke H, Klamt J, Hofmann A, Gölz L, Raff R et al (2016) Sequencing the GRHL3 coding region reveals rare truncating mutations and a common susceptibility variant for nonsyndromic cleft palate. Am J Hum Genet 98:755–762 10.1016/j.ajhg.2016.02.013 PMC4833214 27018475

[22] de la Garza G, Schleiffarth JR, Dunnwald M, Mankad A, Weirather JL, Bonde G, Butcher S, Mansour TA, Kousa YA, Fukazawa CF et al (2013) Interferon regulatory factor 6 promotes differentiation of the periderm by activating expression of Grainyhead-like 3. J Invest Dermatol 133:68–77 10.1038/jid.2012.269 PMC3541433 22931925

[23] Carroll SH, Macias Trevino C, Li EB, Kawasaki K, Myers N, Hallett SA, Alhazmi N, Cotney J, Carstens RP, Liao EC (2020) An Irf6-Esrp1/2 regulatory axis controls midface morphogenesis in vertebrates. Development. 10.1242/dev.194498 PMC7774891 33234718

[24] Hall EG, Wenger LW, Wilson NR, Undurty-Akella SS, Standley J, Augustine-Akpan EA, Kousa YA, Acevedo DS, Goering JP, Pitstick L et al (2020) SPECC1L regulates palate development downstream of IRF6. Hum Mol Genet 29:845–858 10.1093/hmg/ddaa002 PMC7104672 31943082

[25] Siewert A, Hoeland S, Mangold E, Ludwig KU (2024) Combining genetic and single-cell expression data reveals cell types and novel candidate genes for orofacial clefting. Sci Rep 14:26492 10.1038/s41598-024-77724-9 PMC11532359 39489835

[26] Siewert A, Reiz B, Krug C, Heggemann J, Mangold E, Dickten H, Ludwig KU (2023) Analysis of candidate genes for cleft lip +/- cleft palate using murine single-cell expression data. Front Cell Dev Biol 11:1091666 10.3389/fcell.2023.1091666 PMC10165499 37169019

[27] Carroll SH, Schafer S, Dalessandro E, Ho TV, Chai Y, Liao EC (2025) Neural crest and periderm-specific requirements of Irf6 during neural tube and craniofacial development. Dev Biol 522:106–115 10.1016/j.ydbio.2025.03.006 PMC12065081 40113028

[28] Dougherty M, Kamel G, Grimaldi M, Gfrerer L, Shubinets V, Ethier R, Hickey G, Cornell RA, Liao EC (2013) Distinct requirements for wnt9a and irf6 in extension and integration mechanisms during zebrafish palate morphogenesis. Development 140:76–81 10.1242/dev.080473 PMC6514306 23154410

[29] Fakhouri WD, Metwalli K, Naji A, Bakhiet S, Quispe-Salcedo A, Nitschke L, Kousa YA, Schutte BC (2017) Intercellular genetic interaction between Irf6 and Twist1 during craniofacial development. Sci Rep 7:7129 10.1038/s41598-017-06310-z PMC5540929 28769044

[30] Thompson J, Mendoza F, Tan E, Bertol JW, Gaggar AS, Jun G, Biguetti C, Fakhouri WD (2019) A cleft lip and palate gene, Irf6, is involved in osteoblast differentiation of craniofacial bone. Dev Dyn 248:221–232 10.1002/dvdy.13 PMC6414085 30684382

[31] Goudy S, Angel P, Jacobs B, Hill C, Mainini V, Smith AL, Kousa YA, Caprioli R, Prince LS, Baldwin S et al (2013) Cell-autonomous and non-cell-autonomous roles for IRF6 during development of the tongue. PLoS ONE 8:e56270 10.1371/journal.pone.0056270 PMC3579850 23451037

[32] Fakhouri WD, Rhea L, Du T, Sweezer E, Morrison H, Fitzpatrick D, Yang B, Dunnwald M, Schutte BC (2012) MCS9.7 enhancer activity is highly, but not completely, associated with expression of *Irf6* and *p63*. Dev Dyn 241:340–349 10.1002/dvdy.22786 PMC4936416 22113860

[33] Zhang J, Hagopian-Donaldson S, Serbedzija G, Elsemore J, Plehn-Dujowich D, McMahon AP, Flavell RA, Williams T (1996) Neural tube, skeletal and body wall defects in mice lacking transcription factor AP-2. Nature 381:238–241 10.1038/381238a0 8622766

[34] Luo T, Lee YH, Saint-Jeannet JP, Sargent TD (2003) Induction of neural crest in *Xenopus* by transcription factor AP2alpha. Proc Natl Acad Sci U S A 100:532–537 10.1073/pnas.0237226100 PMC141030 12511599

[35] Mori-Akiyama Y, Akiyama H, Rowitch DH, de Crombrugghe B (2003) Sox9 is required for determination of the chondrogenic cell lineage in the cranial neural crest. Proc Natl Acad Sci U S A 100:9360–9365 10.1073/pnas.1631288100 PMC170923 12878728

[36] Yankee TN, Oh S, Winchester EW, Wilderman A, Robinson K, Gordon T, Rosenfeld JA, VanOudenhove J, Scott DA, Leslie EJ et al (2023) Integrative analysis of transcriptome dynamics during human craniofacial development identifies candidate disease genes. Nat Commun 14:4623 10.1038/s41467-023-40363-1 PMC10397224 37532691

[37] Feng J, Janeckova E, Guo T, Ziaei H, Zhang M, Geng JJ, Cha S, Araujo-Villalba A, Liu M, Ho TV et al (2025) High-resolution spatial transcriptomics and cell lineage analysis reveal spatiotemporal cell fate determination during craniofacial development. Nat Commun 16:4396 10.1038/s41467-025-59206-2 PMC12069723 40355462

[38] Perez G, Barber GP, Benet-Pages A, Casper J, Clawson H, Diekhans M, Fischer C, Gonzalez JN, Hinrichs AS, Lee CM et al (2025) The UCSC Genome Browser database: 2025 update. Nucleic Acids Res 53:D1243-d1249 10.1093/nar/gkae974 PMC11701590 39460617

[39] Ishii M, Arias AC, Liu L, Chen YB, Bronner ME, Maxson RE (2012) A stable cranial neural crest cell line from mouse. Stem Cells Dev 21:3069–3080 10.1089/scd.2012.0155 PMC3495126 22889333

[40] Liu Z, Gong B, Li X, Zhao C, Chen Y, Li S, Abduwali R, Bai Y, Teng L, Lu J (2024) Global research trends and focuses on Pierre Robin sequence from 1992 to 2023: a bibliometric analysis of the past 3 decades. J Craniofac Surg 35:1631–1636 10.1097/SCS.0000000000010186 38830018

[41] Bhatia S, Gordon CT, Foster RG, Melin L, Abadie V, Baujat G, Vazquez MP, Amiel J, Lyonnet S, van Heyningen V et al (2015) Functional assessment of disease-associated regulatory variants in vivo using a versatile dual colour transgenesis strategy in zebrafish. PLoS Genet 11:e1005193 10.1371/journal.pgen.1005193 PMC4452300 26030420

[42] Weider M, Wegener A, Schmitt C, Küspert M, Hillgärtner S, Bösl MR, Hermans-Borgmeyer I, Nait-Oumesmar B, Wegner M (2015) Elevated in vivo levels of a single transcription factor directly convert satellite glia into oligodendrocyte-like cells. PLoS Genet 11:e1005008 10.1371/journal.pgen.1005008 PMC4334169 25680202

[43] Lim F, Solvason JJ, Ryan GE, Le SH, Jindal GA, Steffen P, Jandu SK, Farley EK (2024) Affinity-optimizing enhancer variants disrupt development. Nature 626:151–159 10.1038/s41586-023-06922-8 PMC10830414 38233525

[44] Fraser FC (1976) The multifactorial/threshold concept – uses and misuses. Teratology 14:267–280 10.1002/tera.1420140302 1033611

[45] Yamaguchi H, Tran LT, Bi J, Urayama A, Yutzey KE, Mishina Y, Komatsu Y (2025) Enhanced BMP signaling in cranial neural crest cells induces aberrant chondrogenesis by upregulating Tbx20 expression during craniofacial development. Biochem Biophys Res Commun 765:151834 10.1016/j.bbrc.2025.151834 40273622

[46] Schneider H, Hadj‐Rabia S, Berking C, Weider M, Steffann J, Rieker RJ, Gölz L, Peschel N (2026) Deep phenotyping and molecular elucidation of a new syndrome: ectodermal dysplasia caused by IRF6 variants. Exp Dermatol. 10.1111/exd.70331 PMC13392586 42487474

[47] Welzenbach J, Hammond NL, Nikolic M, Thieme F, Ishorst N, Leslie EJ, Weinberg SM, Beaty TH, Marazita ML, Mangold E et al (2021) Integrative approaches generate insights into the architecture of non-syndromic cleft lip with or without cleft palate. HGG Adv 2:100038 10.1016/j.xhgg.2021.100038 PMC8756534 35047836

[48] Danielian PS, Muccino D, Rowitch DH, Michael SK, McMahon AP (1998) Modification of gene activity in mouse embryos in utero by a tamoxifen-inducible form of Cre recombinase. Curr Biol 8:1323–1326 10.1016/s0960-9822(07)00562-3 9843687

[49] Srinivas S, Watanabe T, Lin CS, William CM, Tanabe Y, Jessell TM, Costantini F (2001) Cre reporter strains produced by targeted insertion of EYFP and ECFP into the ROSA26 locus. BMC Dev Biol 1:4 10.1186/1471-213X-1-4 PMC31338 11299042

[50] Gehlen-Breitbach S, Schmid T, Fröb F, Rodrian G, Weider M, Wegner M, Gölz L (2023) The Tip60/Ep400 chromatin remodeling complex impacts basic cellular functions in cranial neural crest-derived tissue during early orofacial development. Int J Oral Sci 15:16 10.1038/s41368-023-00222-7 PMC10079831 37024457

