# Supplementary material for: The orofacial cleft risk gene IRF6 is a target gene of SOX9 in cranial neural crest cells
Source: J Mol Med (Berl). 2026 Sep 26;104(1):113. doi: 10.1007/s00109-026-02720-7 (PMC13615914; doi:10.1007/s00109-026-02720-7)
Supplement: Supplementary file 1 [file 109_2026_2720_MOESM1_ESM.pdf]

Supplementary methods

Cloning procedures

For cloning of MCS-9.7, it was amplified from human genomic DNA (Promega) with the PCR primers listed in Supplementary Table 4 that contained restriction sites for SacI and XhoI, respectively. The PCR fragment was separated by agarose gel electrophoresis, purified with QIAquick PCR & gel cleanup kit (Qiagen), restriction digested with SacI and XhoI and cloned upstream of a beta-globin minimal promoter into pGL2 (Promega) that had been cut with the same enzymes. The ligation reaction was transformed into *E. coli* NEB 5-alpha (New England Biolabs). Single colonies were picked for amplification of plasmids. Correctness of cloned DNA sequence was proven by Sanger sequencing. This procedure yielded the ancestral allele of rs76145088.

In order to receive the derived allele of rs76145088, MCS-9.7 was subcloned into a standard cloning vector with SacI and XhoI. The resulting plasmid was used as template for site-directed mutagenesis with Q5 site-directed mutagenesis kit (New England Biolabs) with the PCR primers listed in Supplementary Table 4. After proof of correct sequence by Sanger sequencing, MCS-9.7 containing the derived allele was cloned into pGL2 upstream of the beta-globin minimal promoter with SacI and XhoI.

As negative control for effector plasmids in luciferase assays, a vector containing the same vector backbone as pCMV6-ENTRY (OriGene) without an open reading frame (ORF) downstream of the CMV promoter/enhancer was derived by removing the ORF with restriction digest with BamHI, followed by Klenow end fill-in and digest with SmaI and religation. Sequence of the resulting pCMV6-ctr was verified by Sanger sequencing.

An expression plasmid for the amino-terminal fragment of SOX9 around dimerization and DNA binding domain (amino acids 1-186) was generated by PCR with primers listed in supplementary table 4 and with pCMV6-ENTRY-SOX9 (OriGene) as template. The PCR product was digested with EcoRI and XhoI and ligated into pCMV6-ENTRY-SOX9 in which the *SOX9* coding sequence had been removed by restriction digest with EcoRI and XhoI.

For cloning of shRNA plasmids targeting human *IRF6* or murine *Sox9* and the scrambled controls (SCR), annealed DNA oligonucleotides (Table S4) were cloned into pSUPER.neo+EGFP that had been cut with HindIII and BglII. Correct sequences were proven by Sanger sequencing.

For sequence analyses, the sequences of the UCSC genome browser were used as reference (https://genome.ucsc.edu/) [1].

Cell culture

The O9-1 cell line was purchased from Sigma-Aldrich and cultured essentially as described [2, 3]. Briefly, cells were seeded on Matrigel (Corning) coated cell culture plates. Cells were maintained in STO-conditioned DMEM (Gibco) containing 15% fetal calf serum (FCS), 1% Penicillin/Streptomycin, 0.1 mM non-essential amino acids and 55 µM beta-mercaptoethanol to which 25 ng/ml bFGF and 5 ng/ml LIF had been added. Medium was changed every second day and passaging was performed with Accumax solution (Sigma-Aldrich) at a confluence of up to 75% with a reseeding density of 1:40.

HaCaT cells (CLS GmbH), HEK293T cells (DSMZ) and Neuro-2a cells (DSMZ) were seeded on standard cell culture plasticware and were kept in DMEM high glucose with GlutaMAX, pyruvate (Gibco), 1% Penicillin/Streptomycin and 10% FCS.

Luciferase assays

For luciferase assays, Neuro-2a cells (DSMZ) grown in 24 well plates were transfected with Superfect (Qiagen) with 750 ng reporter and 750 ng effector plasmid per triplicate. Effector plasmids containing coding sequences for human *SOX9* or mouse *Tfap2a* were based on pCMV6-ENTRY (OriGene) or pCMV5, respectively. In case of reduced amounts of effector plasmids or negative controls, empty vector pCMV6-ctr was added to a total plasmid amount of 1.5 µg. 48 h after transfection, whole cell extracts were prepared, and luciferase activities were determined in the presence of luciferin and ATP 48 h in a Synergy H1 plate reader (BioTek). SOX9- and TFAP2A-dependent fold activations were calculated by arbitrarily setting the relative luciferase units in the absence of effector to 1.

For analysis of activation of MCS-9.7 by endogenous transcription factors, O9-1 or HaCaT cells were seeded in 24 well plates and transfected with Lipofectamine 3000 (Invitrogen) according to the manufacturer’s instructions. To knockdown endogenous Sox9 levels, pSUPER plasmids carrying shRNAs targeting murine *Sox9*, pSUPER plasmids carrying a scrambled shRNA or empty pSUPER were co-transfected with reporter plasmids in 1:5 ratio.

In order to compare activation of MCS-9.7 across different cells lines, transfections with a plasmid containing the luciferase ORF under control of a CMV enhancer/promoter in 1/100 amount (and 99/100 empty pCMV5) were carried out in parallel. Relative luciferase units (RLUs) of MCS-9.7 were set into relation to the RLUs of CMV-based plasmids.

Generation of CRISPR/Cas9 knockout clones

We generated *Sox9*-deficient O9-1 cell lines by CRISPR/Cas9-mediated genome editing according to an established strategy in our laboratory [2]. Briefly, guide sequences (see Fig. 3A) that had been designed with the CRISPOR online server (Version 4.99) [4] were cloned as annealed DNA oligonucleotides into pX330-U6-chimeric_BB-CBh-hSPCas9

(obtained from Addgene, plasmid #42230; oligonucleotide sequences are listed in Supplementary Table 4) [5] that had been cut with BbsI. After co-transfection with pEGFP-N1 (Clontech) and 3 d of culture, EGFP-positive cells were FACS-sorted to single cells. Loss of *Sox9* expression in clonal lines was analyzed by immunofluorescence and Western blot. Genomic DNA of clonal lines was PCR-amplified around the target site (primer sequences given in Supplementary Table 4), sequenced and checked for successful editing with Synthego ICE analysis 2019, v3.0 and by manual comparison of sequenced DNA with original genomic sequence.

Immunofluorescent stainings

For immunofluorescent stainings on tissue sections, mouse embryos were obtained at E9.5, E10.5 and E11.5 and fixed in PBS with 4% paraformaldehyde for 3–4 h, washed with PBS, incubated for 7 days at 4 °C in PBS containing 30% sucrose, embedded in tissue freezing medium (Leica) and stored at −80 °C until further use. 10 μm sections were produced on a Leica cryotome CM3050 S, placed on glass slides, washed, permeabilized with 0.5% Triton X-100 and incubated in blocking solution (PBS with 10% fetal calf serum, 1% bovine serum albumin and 0.5% Triton X-100) for 2 h at RT. Sections were incubated with primary antibodies in blocking buffer at 4°C over night (for antibodies see Supplementary Table 3), followed by incubation with secondary antibodies (Alexa-488- or Cy-3-coupled) in blocking buffer for 2 h at room temperature. YFP signal was enhanced by labelling with an anti-GFP antibody. Sections were counterstained with Hoechst 33342 and mounted with Mowiol 4-88 (Carl Roth). Image acquisition was performed on a Leica Mica widefield microscope.

For quantification of Irf6 signal intensities, the software QuPath (v. 0.5.0) was used [6]. First, regions of epithelial cells and of CNCCs were defined in each section. Nuclei were identified automatically in DAPI channel. Irf6 signal intensity was determined as mean intensity for each nucleus. From mean values for single nuclei, the mean values for each region were calculated in Microsoft Excel 16. For each embryo, at least three sections were analyzed and of these, the mean was determined. At least three embryos were analyzed per embryonic stage. In order to determine Irf6 signal ratio in CNCCs to epithelial cells, the mean CNCC signal was divided by the mean epithelial signal for each section and means of these ratios were determined. For quantification of $Irf6^{high}$ cells, the percentage of cells among CNCCs with an Irf6 nuclear signal intensity equal to or higher than the mean Irf6 signal intensity in epithelial cells of the respective section was determined.

For immunocytochemistry, O9-1 cells were seeded on Matrigel-coated and HCl-treated glass coverslips; HaCaT cells were seeded on 4-chamber slides, PCA (Sarstedt). Cells were fixed (three days after transfection, if applicable) in PBS with 4% paraformaldehyde for 10 min and washed with PBS. Permeabilization, blocking and staining procedures were performed as

described above but with shorter incubation times (blocking, primary and secondary antibody incubation: 1 h each) and with DAPI as nuclear counterstain. Quantification of IRF6 signal intensity was carried out as described above, with setting the nuclear IRF6 signal intensities of transfected cells in relation to the mean nuclear IRF6 signal intensitiy of non-transfected cells in the same image section.

RNA extraction, qRT-PCR and Western blot

RNA was harvested from cells in logarithmic growth phase with the RNeasy plus kit (Qiagen) according to manufacturer's instructions. RNA was reverse transcribed with SCRIPT cDNA synthesis kit (Jena Bioscience). The qRT-PCR reaction was performed on a Roche LightCycler 96 with the primer pairs listed in Supplementary Table 4. Quantification was performed by the ΔΔ-Ct method with Ct values normalized to the housekeeping gene *Rplp0*. As positive control for *Grhl3* expression, O9-1 cells transfected with *Grhl3* expression plasmid (Addgene #172869) were used [7].

Western blots were performed as described [2] with the exception that total protein lysates were prepared with CelLytic buffer (Sigma-Aldrich). Equal amounts of total protein as determined by DC assay were loaded onto gels. Detection was performed with a ChemiDoc imaging system (Bio-Rad). Size of detected proteins was estimated from co-electrophoresed protein size marker (Invitrogen). Quantification of protein levels was performed with Bio-Rad Image Lab 6.1.

Electrophoretic mobility shift assay and chromatin immunoprecipitation

EMSA was performed with biotinylated double-stranded oligonucleotides encompassing the putative SOX9 binding site spanning rs76145088 as listed in table S4. Protein extracts from HEK293T cells that had been transfected with expression plasmids for the amino-terminal fragment of SOX9 (amino acids 1-186) encompassing dimerization domain and DNA binding domain or with EGFP as negative control were used for EMSA. Cell extracts were incubated on ice with 100 nM oligonucleotides in the presence of poly-dG•dC (InvivoGen) [8], bands were separated on a 6% polyacrylamide DNA retardation gel (Thermo Fisher) in 0.5x TBE buffer. DNA was blotted onto a nylon membrane and cross-linked with UV light on a ChemiDoc device (Bio-Rad). After blocking with BSA, membranes were incubated with Streptavidin-HRP (Thermo Fisher). Detection was performed with Clarity Western ECL (Bio-Rad) on a ChemiDoc device (Bio-Rad). Quantification of band intensities was performed with Bio-Rad Image Lab 6.1. Intensities of shifted probe were set into relation to the sum of intensities of shifted probe and free probe of each lane.

ChIP was performed with the SimpleChIP Enzymatic Chromatin IP Kit (Magnetic Beads) (New England Biolabs) according to the manufacturer's instructions with the exception that

chromatin was sheared by sonication with a Bioruptor (Diagenode). Rabbit antiserum directed against Sox9 (Invitrogen, table S3) and rabbit IgG (New England Biolabs) were used for precipitation of chromatin. Detection of precipitated DNA was performed with qPCR on a Roche LightCycler 96 with the primer pairs listed in Supplementary Table 4. The amount of DNA precipitated with Sox9 antiserum was set into relation to the amount of DNA precipitated with IgG for each experiment and the mean was calculated from these ratios.

1. Perez G, Barber GP, Benet-Pages A, Casper J, Clawson H, Diekhans M, Fischer C, Gonzalez JN, Hinrichs AS, Lee CM, et al. (2025) The UCSC Genome Browser database: 2025 update. Nucleic Acids Res 53: D1243-d1249. DOI 10.1093/nar/gkae974
2. Gehlen-Breitbach S, Schmid T, Fröb F, Rodrian G, Weider M, Wegner M, Gölz L (2023) The Tip60/Ep400 chromatin remodeling complex impacts basic cellular functions in cranial neural crest-derived tissue during early orofacial development. International Journal of Oral Science 15: 16. DOI 10.1038/s41368-023-00222-7
3. Ishii M, Arias AC, Liu L, Chen YB, Bronner ME, Maxson RE (2012) A stable cranial neural crest cell line from mouse. Stem Cells Dev 21: 3069-3080. DOI 10.1089/scd.2012.0155
4. Concordet JP, Haeussler M (2018) CRISPOR: intuitive guide selection for CRISPR/Cas9 genome editing experiments and screens. Nucleic Acids Res 46: W242-w245. DOI 10.1093/nar/gky354
5. Cong L, Ran FA, Cox D, Lin S, Barretto R, Habib N, Hsu PD, Wu X, Jiang W, Marraffini LA, et al. (2013) Multiplex genome engineering using CRISPR/Cas systems. Science (New York, NY) 339: 819-823. DOI 10.1126/science.1231143
6. Bankhead P, Loughrey MB, Fernández JA, Dombrowski Y, McArt DG, Dunne PD, McQuaid S, Gray RT, Murray LJ, Coleman HG, et al. (2017) QuPath: Open source software for digital pathology image analysis. Scientific Reports 7: 16878. DOI 10.1038/s41598-017-17204-5
7. Mourao L, Zeeman AL, Wiese KE, Bongaarts A, Oudejans LL, Martinez IM, van de Grift YBC, Jonkers J, van Amerongen R (2021) Hyperactive WNT/CTNNB1 signaling induces a competing cell proliferation and epidermal differentiation response in the mouse mammary epithelium. bioRxiv: 2021.2006.2022.449461. DOI 10.1101/2021.06.22.449461
8. Weider M, Wegener A, Schmitt C, Küspert M, Hillgärtner S, Bösl MR, Hermans-Borgmeyer I, Nait-Oumesmar B, Wegner M (2015) Elevated in vivo levels of a single transcription factor directly convert satellite glia into oligodendrocyte-like cells. PLoS genetics 11: e1005008. DOI 10.1371/journal.pgen.1005008
